# The Protective Effect of *Rhizoma Dioscoreae* Extract against Alveolar Bone Loss in Ovariectomized Rats via Regulating Wnt and p38 MAPK Signaling

**DOI:** 10.3390/nu6125853

**Published:** 2014-12-12

**Authors:** Zhiguo Zhang, Lihua Xiang, Dong Bai, Wenlai Wang, Yan Li, Jinghua Pan, Hong Liu, Shaojun Wang, Gary Guishan Xiao, Dahong Ju

**Affiliations:** 1 Institute of Basic Theory, China Academy of Chinese Medical Sciences, Beijing 100700, China; E-Mails: zzgtcm@163.com (Z.Z.); xlh891201@sina.com (L.X.); abai2000_0@163.com (D.B.); wangwenlai666@163.com (W.W.); lei_ruo@163.com (Y.L.); jh-p@163.com (J.P.); liuhong@163.com (H.L.); wangshaojun@163.com (S.W.); 2 School of Pharmaceutical Science, Dalian University of Technology, Dalian 116024, China; 3 Functional Genomics and Proteomics Laboratory, Osteoporosis Research Center, Creighton University Medical Center, Omaha, NE 68131, USA

**Keywords:** alveolar bone loss, gene expression profile, herbal medicine, ovariectomized rats, *Rhizoma Dioscoreae*

## Abstract

Aim: The aim of this study was to evaluate the osteoprotective effect of aqueous *Rhizoma Dioscoreae* extract (RDE) on the alveolar bone of rats with ovariectomy-induced bone loss. Methods: Female Wistar rats were subjected to either ovariectomy or a sham operation (SHAM). The ovariectomized (OVX) rats were treated with vehicle (OVX) or RDE by oral gavage or with 17β-estradiol (E2) subcutaneously. After treatments, the bone mineral density (BMD), the three-dimensional bone architecture of the alveolar bone and the plasma biomarkers of bone turnover were analyzed to assess bone metabolism, and the histomorphometry of the alveolar bone was observed. Microarrays were used to evaluate gene expression profiles in alveolar bone from RDE-treated and OVX rats. The differential expression of genes was further analyzed using Ingenuity Pathway Analysis (IPA). The key findings were verified using real-time quantitative RT-PCR (qRT-PCR). Results: Our results showed that RDE inhibited alveolar bone loss in OVX rats. Compared to the OVX rats, the RDE-treated rats showed upregulated expression levels of 207 genes and downregulated expression levels of 176 genes in the alveolar bone. The IPA showed that several genes had the potential to code for proteins that were involved in the Wnt/β-catenin signaling pathway (*Wnt7a*, *Fzd2*, *Tcf3*, *Spp1*, *Frzb*, *Sfrp2* and *Sfrp4*) and the p38 MAPK signaling pathway (*Il1rn* and *Mapk14*). Conclusion: These experiments revealed that RDE could inhibit ovariectomy-induced alveolar bone loss in rats. The mechanism of this anti-osteopenic effect in alveolar bone may be involved in the reduced abnormal bone remodeling, which is associated with the modulation of the Wnt/β-catenin and the p38 MAPK signaling pathways via gene regulation.

## 1. Introduction

Bone remodeling is a lifelong dynamic process in which mature bone tissue is removed from the skeleton and new bone tissue is formed. Abnormal bone metabolism is due to imbalanced bone formation and resorption, which often occurs in estrogen-deficient ovariectomized (OVX) rats and postmenopausal women [1,2]. Alveolar bone is essential for the support of teeth, which are anchored to the bone by desmodontal fibers. Previous research has shown that osteoporosis may reduce alveolar bone mass and alter alveolar bone structure [3]. This bone loss occurs rapidly during the early postmenopausal period and levels off approximately six years after menopause, likely due to the decrease in estrogen production in postmenopausal women [4]. There exists a relationship between alveolar bone loss and osteoporosis in postmenopausal women [5]. The study showed that alveolar bone loss was significantly correlated with the bone mineral density (BMD) of the trochanter, Ward’s triangle and femur of postmenopausal women [6]. Postmenopausal women with osteoporosis have a greater chance of having alveolar bone loss than do those without osteoporosis [7]. Alveolar bone loss leads to mobile teeth or tooth loss, which seriously reduce the quality of life of postmenopausal women [8].

Estrogen [9], bisphosphonates [10] or parathyroid hormone (PTH) [11] have been used to prevent postmenopausal alveolar bone loss, but many lines of evidence indicate that long-term treatments with those drugs might cause adverse reactions, such as an increased risk of ovarian and endometrial cancer [12,13], osteonecrosis of the jaws [14], nervous system disorders [15] and venous thromboembolism [16]. Thus, an alternative therapeutic strategy with proven efficacy and safety should be developed to prevent and treat alveolar bone loss. For years, Chinese herbal medicine has been widely used in clinical practice to treat bone diseases and will most likely continue to be used as a cost-effective alternative medicine in China [17]. *Rhizoma Dioscoreae* (RD) is the dried rhizome of *Dioscorea opposite* Thunb*.* and has been used to strengthen bone for a long time in China. Our previous studies indicated that treatment with *Rhizoma Dioscoreae* extract (RDE) protects against bone loss of the peripheral skeleton in ovariectomized (OVX) rats [18], a model of postmenopausal osteoporosis. This led us to question whether RDE had a similar protective effect in alveolar bone loss, though the alveolar bone was morphologically and functionally different from other bones of the axial or peripheral skeleton. The aims of this study were to analyze the anti-osteopenic effect of RDE on alveolar bone in OVX rats and to explore the molecular targets of RDE.

## 2. Experimental Section

### 2.1. Preparation of Aqueous Extract

The preparation of RDE was performed as we previously reported [18]. In this study, we used the same batch of RDE as presented previously.

### 2.2. Animal Grouping and Treatments

Many studies have used 6-month-old female rats that have undergone a bilateral ovariectomy to model postmenopausal osteoporosis [19,20]. We obtained a total of forty-eight 6-month-old virgin Wistar rats with a body weight of 310 ± 20.0 g from the Experimental Animal Center of the Academy of Military Medical Sciences (SCXK-(Military) 2002-001, Beijing, China). The Institutional Ethics Committee of the China Academy of Chinese Medical Sciences approved the experimental research on the animals (Approval Number 2012-006). The acclimatized rats were either sham-operated (SHAM, *n* = 12) or bilaterally OVX (*n* = 36) using the dorsal approach [21]. The OVX rats were randomly divided into three groups: OVX group (OVX, *n* = 12); 17β-estradiol treatment group (E2, *n* = 12); RDE group (RDE, *n* = 12). 17β-Estradiol (Sigma-Aldrich, Saint Louis, MO, USA) was prepared by dissolving a small amount in ethanol and adjusting the volume using olive oil. The rats in the E2 group were treated with daily subcutaneously administered 17β-estradiol (30 μg/kg body weight). The rats in the RDE group were treated with RDE dissolved in distilled water at 1.3 g/kg body weight/day by oral gavage. The gavage dosage was based on the recommended dosage for humans (30 g/day) according to the Chinese Pharmacopeia, adjusted for the rat/human body mass ratio. The rats in the SHAM and the OVX groups were administered the same volume of distilled water by oral gavage. All rats were fed standard chow during the course of the experiments (Animal Center of the Fourth Military Medical University, Xi’an, China). All treatments started 1 week after surgery and continued for 12 weeks.

### 2.3. Preparation of Specimens

The day after the last treatment, the animals were anesthetized with an intraperitoneal injection of ketamine (80 mg/kg body weight) and xylazine (12 mg/kg body weight) and sacrificed by exsanguination. We obtained blood samples by puncturing the abdominal aorta before death; we collected the blood samples in heparinized tubes. The blood samples were centrifuged at 3000× *g* at 4 °C for 10 min, aliquoted and frozen at −80 °C until the samples were used for assay. The right mandibles were dissected, filled with physiological saline and stored at −20 °C for measurements of BMD and microstructure by micro-computerized tomography (micro-CT). After the measurement of micro-CT, the right mandibles were used for histological observation. The left mandibles were dissected and stored at −80 °C for microarray and real-time quantitative RT-PCR (qRT-PCR) assays.

### 2.4. Biochemical Markers of Bone Turnover

The plasma levels of bone formation marker, procollagen type 1 *N*-terminal propeptide (P1NP), and bone resorption marker, *C*-terminal cross-linked telopeptides of type I collagen (CTX), were assessed using the enzyme-linked immunosorbent assay (Immunodiagnostic Systems Ltd., Boldon, UK) for control, standard and duplicate tests. Absorbance was read using an ELISA reader (Bio-Tek, Colmar, France) at 450 nm.

### 2.5. Micro-CT Analysis

The right mandible of each animal, without sample preparation or decalcification, was scanned with a high-resolution micro-CT (SkyScan 1172 micro-CT system, Antwerp, Belgium). The SkyScan micro-CT uses the cone-beam reconstruction method to determine the conical geometry of the X-ray source. The desktop SkyScan micro-CT system was used according to a method previously described [22]. Each sample was placed on the rotational stage and translated along the continuously variable magnification stage to achieve the desired resolution (6.8 μm). The sample was rotated 185°, and the images were obtained every 0.9°. Repeated scans were performed at the beginning of the experiment to verify the reproducibility of the method. A low-pass filter was used to remove noise from the resulting gray-scale images. The trabecular bone was determined by a fixed threshold.

After the images were captured (100 keV, 100 μA), we used CTanalyser, an image analysis software of SkyScan, to establish a square region of interest (ROI) (1 mm × 1 mm) on sagittal images of the first molar. All selected ROIs had no overlapping areas with tooth roots. A cubic region (1 mm × 1 mm × 1 mm) beginning 1.5 mm beneath the lowest point of the first molar crown was established as the “volume of interest” (VOI) using NRecon, a three-dimensional 3D reconstruction software from SkyScan. We performed morphological measurements of the trabecular bone within the VOI using the standard SkyScan software package. We used three-dimensional analyses to assess the BMD, bone volume fraction (BV/TV), the trabecular thickness (Tb.Th), the trabecular separation (Tb.Sp), the trabecular number (Tb.N), the structural model index (SMI) and the degree of anisotropy (DA) for the same VOI [23].

### 2.6. Histological Observation

The right mandibles were fixed in 10% buffered formalin, decalcified in 14% EDTA, dehydrated and embedded in paraffin. Sections were cut using a standard microtome (Leica DMB6000B and CTR6000, Leica, Wetzlar, Germany), affixed to glass slides and stained with hematoxylin and eosin.

### 2.7. Microarray Data Analysis

Alveolar bone was prepared from six RDE rats and six OVX rats. The gene microarray assay was conducted by KangChen Bio-tech (Shanghai, China). We performed a microarray analysis of whole-genome gene expression profiling using Agilent chips (4 × 44 K, Agilent Technologies), which consisted of approximately 41,000 probes for rat genes. The total RNA of distal right alveolar bone from the RDE group and OVX group was harvested using TRIzol (Invitrogen, Carlsbad, CA, USA) and the RNeasy kit (Qiagen, Chatsworth, CA, USA), according to the manufacturer’s instructions, including a DNase digestion step. After RNA measurement using the NanoDrop ND-1000 (NanoDrop Technologies, Montchanin, DE, USA)and denaturing gel electrophoresis, the samples were amplified and labeled using an Agilent Quick Amp labeling kit and hybridized with an Agilent whole genome oligo microarray in Agilent’s Sure-Hyb Hybridization Chambers (Agilent Technologies, Santa Clara, CA, USA). After hybridization and washing, the processed slides were scanned with the Agilent DNA microarray scanner using settings recommended by Agilent Technologies. The resulting text files extracted from Agilent Feature Extraction Software were imported into Agilent GeneSpring software (Agilent, version 11.0, Agilent Technologies, Santa Clara, CA, USA) for further analysis. The gene expression was normalized to the OVX group. The differentially expressed genes were identified through the fold change and *t*-test *p*-value screening.

### 2.8. Ingenuity Pathway Analysis

To facilitate the gene microarray data analysis and to relate specific genes to the underlying biological processes, Ingenuity Pathway Analysis (IPA) (Ingenuity^®^ Systems, Redwood, CA, USA) was used for the function and pathway analysis. Differentially expressed genes between the RDE group and the OVX group were imported into IPA, and the top five canonical pathways were observed. The canonical pathway analysis identified the molecular pathways from the IPA library of canonical pathways that were most significant to the dataset. The genes from the dataset that were associated with a canonical pathway were considered for additional analysis.

### 2.9. Quantitative Real-Time RT-PCR (qRT-PCR)

The total RNA was purified using an RNeasy Mini Kit (Qiagen, Valencia, CA, USA), and 4 μg RNA was reverse-transcribed using the Superscript First Strand synthesis system (Invitrogen, Carlsbad, CA, USA) to cDNA. The qRT-PCR amplification was performed using the SYBR-green detection of PCR products in real time with an ABI-7500 Sequence Detection System (Applied Biosystems, Foster City, CA, USA) according to the manufacturer’s instruction. The primers used in the qRT-PCR analysis are presented in Table 1. The PCR program was initiated by 10 s at 95 °C before 40 thermal cycles, each of 5 s at 95 °C and 34 s at 60 °C. We analyzed the data according to the 2^−^^∆∆Ct^ method and normalized to the glyceraldehyde-3-phosphate dehydrogenase (GAPDH) expression in each sample. The melting curves for each PCR reaction were generated to ensure the purity of the amplification product. A no-template negative control was included in each experiment.

**Table 1:** Primers used for qRT-PCR analysis.

Transcript	Sequence (5′–3′)
*Gapdh*	F: 5′-GGGAAACTGTGGCGTGAT-3′ R: 5′-GAGTGGGTGTCGCTGTTGA-3′
*Fzd2*	F: 5′-CAGGGCACTAAGAAAGAAGGCT-3′ R: 5′-AGGAACCAGGTGAGGGACAGA-3′
*Il1rn*	F: 5′-CTTACCTTCATCCGCTCCGA-3′ R: 5′-GATCAGGCAGTTGGTGGTCAT-3′
*Mapk14*	F: 5′-ATAGACGAATGGAAGAGCCTGAC-3′ R: 5′-CAAAGATACATGGACAAACGGAC-3′
*Sfrp2*	F: 5′-CCGAAAGGGACCTGAAGAAAT-3′ R: 5′-ACCAGATACGGAGCGTTGATG-3′
*Sfrp4*	F: 5′-AAGTCTTTGTCACCTATCCCTCG-3′ R: 5′-CGGCTGGCTATCTGCTTCTT-3′
*Spp1*	F: 5′-TTTCACTCCAATCGTCCCTACA-3′ R: 5′-AGTCCATAAGCCAAGCTATCACC-3′
*Tcf3*	F: 5′-ACAGTCTCAGCAGCAAATCCAA-3′ R: 5′-GAAGACGCAGGGCTATCACAA-3′
*Ptk2b*	F: 5′-TCTGTGACCCGTCTACCCATC-3′ R: 5′-CTTTCTCCAGCACTCCGATGA-3′
*Wnt7a*	F: 5′-CTCTGCCGACATCCGCTAC-3′ R: 5′-CGACCCGCCTCGTTATTG-3′
*Bmp1*	F: 5′-CAAAGGACCCGACTCAGCA-3′ R: 5′-CCACATAGTCATACCAGCACAGG-3′
*Csf1r*	F: 5′-AAGCCGAAATATCAGGTGCG-3′ R: 5′-GGGTCGATGAAGGTGTAGTTGTT-3′
*Frzb*	F: 5′-TCCAAGGGATACCGTCAACC-3′ R: 5′-ATCCTTCCACTTCTCAGCGATAG-3′

### 2.10. Western Analysis

Alveolar bone was crushed in liquid nitrogen to extract bone proteins and solubilized in radioimmunoprecipitation assay (RIPA) buffer with protease inhibitors and phosphatase inhibitors. Insoluble substances were separated and removed by centrifugation at 10,000 rpm for 5 min at 4 °C. A commercial BCA reagent (Pierce, Rockford, IL, USA) was used to determine protein concentrations. Eighty micrograms of proteins from each sample were resolved, used for electrophoresis on 12% SDS-polyacrylamide gels and transferred onto nitrocellulose membranes (Hybond-ECL; GE Healthcare, Piscataway, NJ, USA). Membranes were blocked with 5% nonfat dry milk for 1 h, incubated overnight at 4 °C with antibodies against Tcf-3 (1:1000 dilution) (Santa Cruz Biotechnology, Santa Cruz, CA, USA), p38α (1:1000 dilution) (Santa Cruz Biotechnology) and β-actin (1:25,000 dilution) (Sigma-Aldrich, St. Louis, MO, USA). Subsequently, membranes were washed and incubated for 1 h using an HRP-linked antibody (1:1000 dilution, Cell signaling Technology, Beverly, MA, USA). For the detection of immunoreactive proteins, an enhanced chemiluminescence kit (PerkinElmer, Waltham, MA, USA) was used. Using Quantity One software (Bio-Rad, Hercules, CA, USA), the intensities of specific bands were quantified with densitometry and then were normalized for β-actin. Normalized data are expressed as the fold increase *vs.* the SHAM or OVX control.

### 2.11. Statistical Analysis

All values were expressed as the mean ± standard deviation. All analyses were conducted using the SPSS 13.0 (SPSS Inc., Chicago, IL, USA). The difference between the groups regarding the evaluated parameters was tested using the analysis of variance (ANOVA) followed by the least significant difference (LSD) test. The data of all groups passed the Kolmogorov–Smirnov test of normality. *p* < 0.05 was considered to be statistically significant.

## 3. Results

### 3.1. Effect of RDE on Biomarkers of Bone Turnover

**Figure 1:**
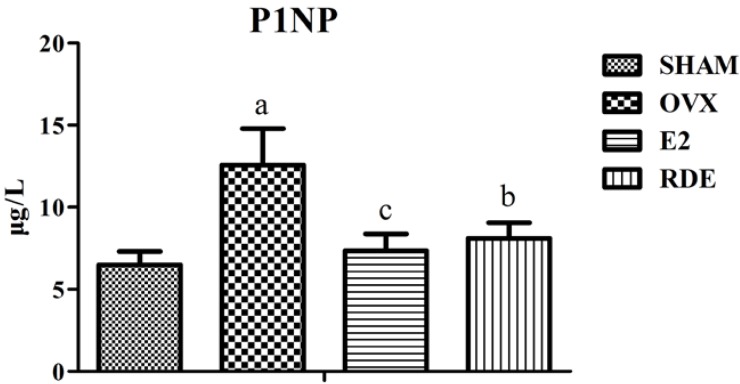
Effect of *Rhizoma Dioscoreae* extract (RDE) on (procollagen type 1 *N*-terminal propeptide) P1NP in plasma after 12 weeks of treatment. ^a^ *p* < 0.01 *vs.* the SHAM group; ^b^ *p* < 0.05 *vs.* the ovariectomized (OVX) group; ^c^ *p* < 0.01 *vs.* the OVX group. E2, 17β-estradiol.

**Figure 2:**
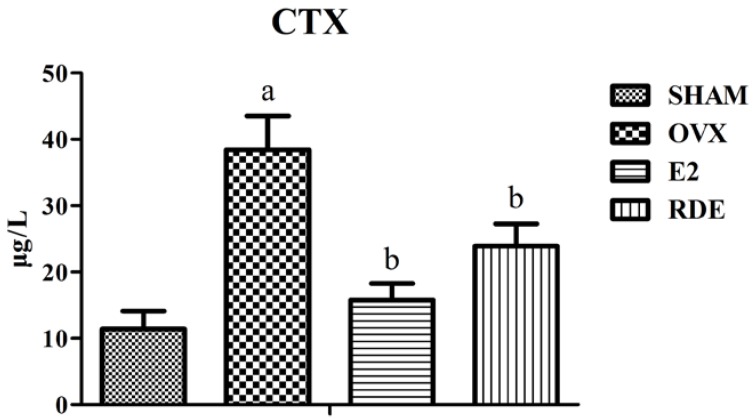
Effect of RDE on *C*-terminal cross-linked telopeptides of type I collagen (CTX) in plasma after 12 weeks of treatment. ^a^ *p* < 0.01 *vs.* the SHAM group; ^b^ *p* < 0.01 *vs.* the OVX group.

The plasma levels of bone formation (procollagen type 1 *N*-terminal propeptide, P1NP) and bone resorption (CTX) biomarkers after a 12-week treatment of different rat groups are provided in Figure 1 and Figure 2. At the end of Week 12, the OVX group showed a significantly higher concentration of P1NP or CTX than the SHAM group (*p* < 0.01). Moreover, the E2 and RDE group showed a significantly lower (*p* < 0.01 or *p* < 0.05) level of P1NP or CTX than the OVX group.

### 3.2. Effect of RDE on BMD and Trabecular Bone Microarchitecture

**Figure 3:**
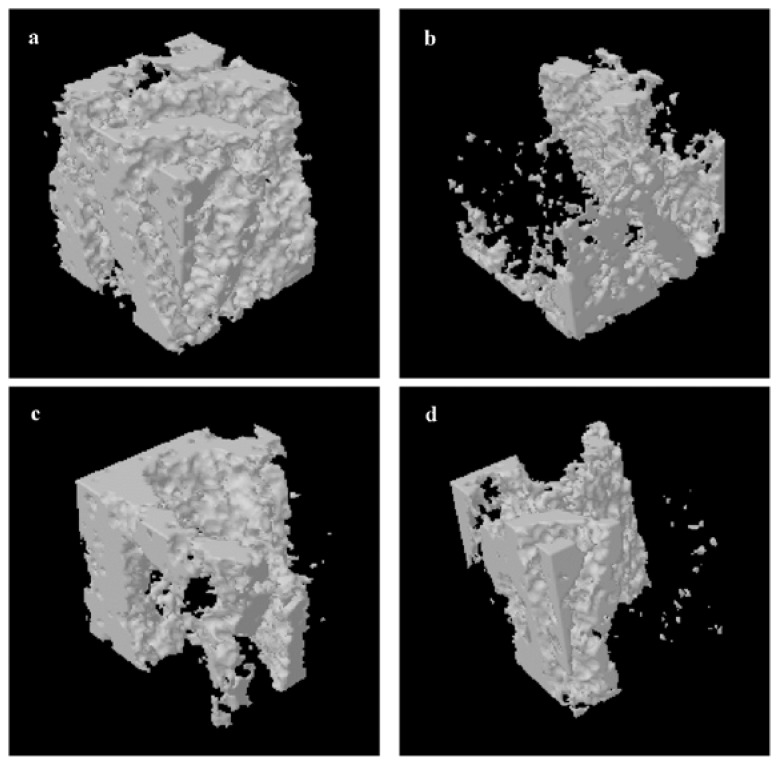
Representative sample from each group: 3D architecture of alveolar bone beneath the lowest point of the first molar crown. (**a**) SHAM; (**b**) OVX; (**c**) E2; (**d**) RDE.

We evaluated the BMD and trabecular bone microarchitecture of four groups. Analysis of the alveolar bone morphometric parameters indicated that ovariectomy significantly decreased the BMD, trabecular BV/TV, Tb.N and Tb.Th (*p* < 0.01) and increased Tb.Sp, SMI and DA (*p* < 0.01) compared to the SHAM group. Treatment with RDE or E2 significantly inhibited the OVX-induced changes (Table 2). Further, treatment with E2 or RDE relieved damage to the trabecula in alveolar bone induced by the ovariectomy (Figure 3a–d).

**Table 2:** Effect of RDE on bone mineral density (BMD) and trabecular bone microarchitecture. BV/TV, bone volume fraction; Tb.Th, trabecular thickness; Tb.Sp, trabecular separation; Tb.N, trabecular number; SMI, structural model index; DA, degree of anisotropy.

Parameters	SHAM	OVX	E2	RDE
BMD (g/cm^3^)	0.348 ± 0.005	0.295 ± 0.006 ^a^	0.328 ± 0.008 ^c^	0.313 ± 0.004 ^b^
BV/TV (%)	55.100 ± 4.718	6.777 ± 4.1569 ^a^	42.756 ± 0.843 ^c^	22.299 ± 2.535 ^c^
Tb.Th (μm)	81.592 ± 8.654	41.237 ± 2.592 ^a^	74.226 ± 6.361 ^c^	49.382 ± 3.976 ^c^
Tb.Sp (μm)	181.62 ± 26.48	678.27 ± 33.21 ^a^	319.63 ± 95.91 ^c^	519.29 ± 13.55 ^c^
Tb.N (1/mm)	6.84 ± 1.28	1.61 ± 0.91 ^a^	5.81 ± 0.61 ^c^	4.52 ± 0.40 ^c^
SMI	0.914 ± 0.275	2.327 ± 0.419 ^a^	1.637 ± 0.217 ^c^	1.712 ± 0.379 ^c^
DA	2.526 ± 0.837	7.770 ± 0.679 ^a^	2.185 ± 0.271	1.861 ± 0.188

Values are presented as the means ± SD (*n* = 12 in each group). ^a^ *p* < 0.01 *versus* the SHAM group; ^b^ *p* < 0.05 *vs.* the OVX group; ^c^ *p* < 0.01 *vs.* the OVX group.

### 3.3. Effect of RDE on Histological Morphology of Alveolar Bone

**Figure 4:**
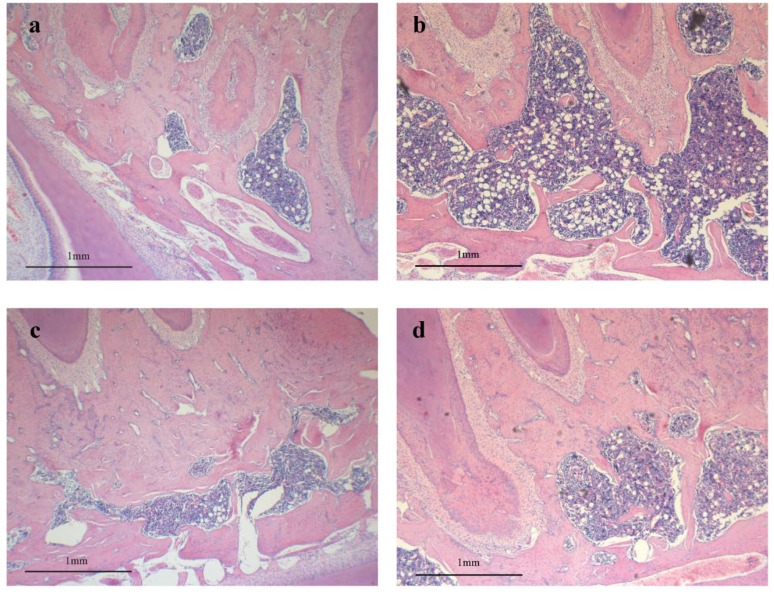
Histological observation of the alveolar bone region of the first molar in rats. The sections were stained with hematoxylin and eosin. (**a**) SHAM; (**b**) OVX; (**c**) E2; (**d**) RDE. Scale bar = 1 mm. Original magnification, 4×.

We performed a histological observation of the alveolar bone region of first molar in rats. Figure 4 shows a representative histological section of the alveolar bone region of the first molar of the sham-operated or OVX rats treated with vehicle, E2 or RDE. Section from rats from with sham operation showed thick alveolar bone with relatively scant marrow space (Figure 4a). OVX markedly reduced alveolar bone volume and increased the extent of the marrow space (Figure 4b). E2 or RDE treatment for 12 weeks markedly increased alveolar bone volume (Figure 4c,d), but E2 had a more potent effect than RDE. The results from micro-CT and histological observation were reciprocal confirmation.

### 3.4. Effect of RDE on Gene Expression Profile

The array results demonstrated that the expression of 383 genes was altered (≥3-fold) between the alveolar bone from the RDE and OVX group rats. Specifically, 207 genes were upregulated, and 176 genes were downregulated. The differentially expressed genes are listed in the Supplementary Materials.

### 3.5. Pathway Analysis of Differentially Expressed Genes

**Figure 5:**
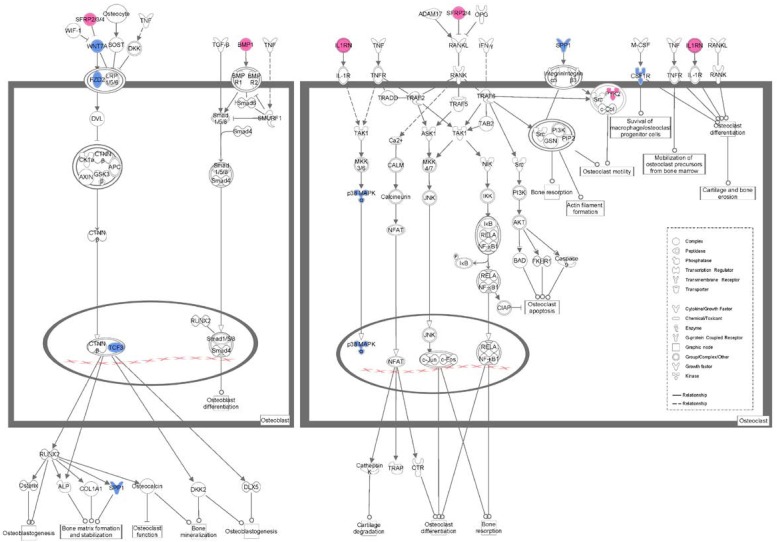
Schematic diagram illustrating the role of osteoblasts and osteoclasts in an anti-osteopenic effect of RDE. The downregulated genes appear in blue, and the upregulated genes appear in red. The white indicates the genes that are not specified, but that are incorporated into the network through relationships.

We characterized differentially expressed genes into functions and signaling pathways by using IPA software according to the Ingenuity Pathways Knowledge Base (IPKB). These signaling pathways were ranked according to the IPA calculated scores, which are based on the significance of the involved genes. Based on IPKB, the signature genes were classified into pathways by IPA. Among these, the top five pathways were identified by IPA with significance values of less than 0.05 (Table 3). Of the top five pathways, two pathways (“role of osteoblasts, osteoclasts and chondrocytes in rheumatoid arthritis” and “Wnt/β-catenin signaling”) were associated with bone metabolism, and the latter pathway was included in the former. In the present study, we selected the following genes, interleukin 1 receptor antagonist (*Il1rn*), secreted frizzled-related protein 2 (*Sfrp2*), secreted frizzled-related protein 4 (*Sfrp4*), wingless-type MMTV integration site family, member 7A (*Wnt7a*), frizzled-related protein (*Frzb*), frizzled family receptor 2 (*Fzd2*), bone morphogenetic protein 1 (*Bmp1*), transcription factor 3 (*Tcf3*), secreted phosphoprotein 1 (*Spp1*, known as osteopontin, *Opn*), mitogen-activated protein kinase 14 (*Mapk14*), colony-stimulating factor 1 receptor (*Csf1r*) and protein tyrosine kinase 2 beta (*Ptk2b*), involved in the two significant signaling pathways associated with bone metabolism (Figure 5), for additional evaluation.

**Table 3:** Top canonical pathways associated with differentially expressed genes between the RDE group and OVX group.

Ingenuity Canonical Pathways	*p*-Value	Number of Molecules
Role of osteoblasts, osteoclasts and chondrocytes in rheumatoid arthritis	1.23 × 10^−3^	12
Role of Wnt/GSK-3β signaling in the pathogenesis of influenza	1.34 × 10^−2^	4
Basal cell carcinoma signaling	2.02 × 10^−2^	4
Human embryonic stem cell pluripotency	2.33 × 10^−2^	6
Wnt/β-catenin signaling	4.07 × 10^−2^	6

### 3.6. Confirmation of Differential Levels of Gene Expression by qRT-PCR

**Figure 6:**
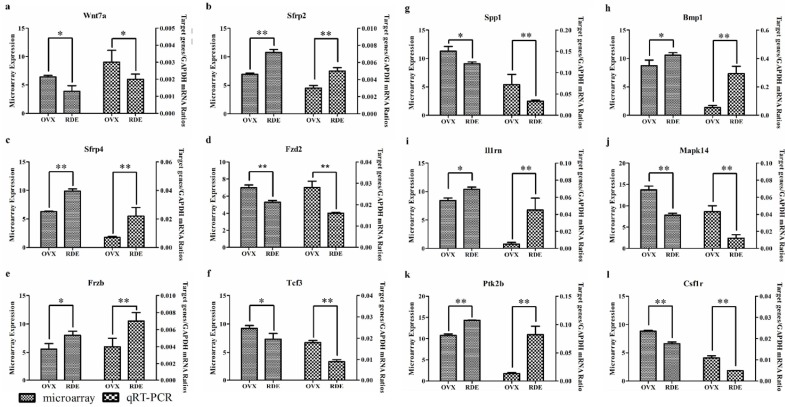
Validation of 12 differentially expressed genes identified by microarray and ingenuity pathway analysis (IPA) in a replicated experiment by qRT-PCR. (**a**)–(**l**) Effect of RDE on the expressions of *Wnt7a*, *Fzd2*, *Tcf3*, *Spp1*, *Mapk14*, *Frzb*, *Sfrp2*, *Sfrp4*, *Il1rn*, *Ptk2b*, *Bmp1* and *Csf1r*, respectively. * *p* < 0.05; ** *p* < 0.01, compared with the OVX group.

To confirm the differential gene expression detected by the microarray analysis and pathway analysis, we sampled 12 genes (*Il1rn*, *Sfrp2*, *Sfrp4*, *Wnt7a*, *Frzb*, *Fzd2*, *Bmp1*, *Tcf3*, *Spp1*, *Mapk14*, *Csf1r* and *Ptk2b*) involved in the significant signaling pathway “role of osteoblasts, osteoclasts and chondrocytes in rheumatoid arthritis” and “Wnt/β-catenin signaling” for verification using qRT-PCR. Figure 6 presents comparative changes in these genes as determined by microarray and qRT-PCR. In all of these cases, the results from qRT-PCR generally agreed with the changes in the microarray analysis, although the absolute degree of change differed markedly between both methods.

### 3.7. Confirmation of Proteins by Western Blotting

**Figure 7:**
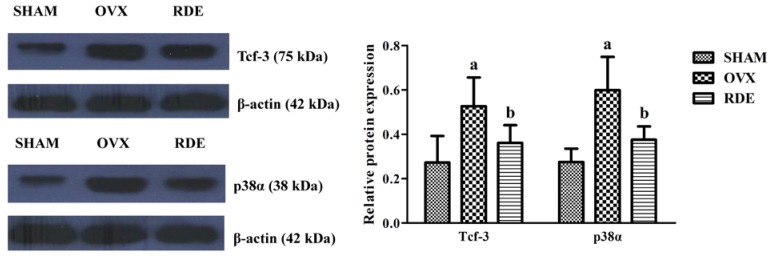
Validation of two proteins by western blotting: Effect of RDE on the expressions of Tcf-3 and p38α. Actin was employed as the housekeeping gene. Representative blots are shown (**left**). Band intensities were quantified from six independent samples using densitometry (**right**). ^a^ *p* < 0.01 *vs.* the SHAM group; ^b^ *p* < 0.01 *vs.* the OVX group.

We further assessed changes in the protein expression of two genes, *Tcf3* and *Mapk14*. We found that changes in the protein expression of Tcf-3 and p38α agreed with the mRNA expression and were downregulated in the RDE-treated rats (Figure 7).

## 4. Discussion

The alveolar bone is a special tissue originating from the dental sac and constantly undergoes chronic inflammatory stimuli or mechanical stress, such as occlusal pressure. Furthermore, the alveolar bone has a very short biological metabolic cycle, and its metabolic mechanism is different from that of other bones to some extent [24]. A large cohort of postmenopausal women who show systemic bone loss and osteoporosis also carry a high risk of tooth and oral bone loss. Such tooth loss and periodontal disease may be associated with alveolar bone resorption [25,26]. Our previous study showed that treatment with RDE protects against bone loss in the peripheral skeleton in OVX rats [18]. However, the effect of RDE on alveolar bone remained unclear. In the present study, we evaluated the effect of RDE on alveolar bone in rats after OVX.

Following ovariectomy, BMD was markedly decreased, due to an increase in alveolar bone turnover in the OVX rats compared to the SHAM rats. In contrast, treatment with the E2 or RDE increased the BMD of the alveolar bone compared to the OVX group.

Coincident and significant increases in the plasma biomarkers for the assessment of bone resorption, CTX, and, for the assessment of bone formation, P1NP indicated that the mature OVX rat is a reliable and suitable animal model for studying high turnover bone loss, such as early postmenopausal osteoporosis. The treatment with E2 or RDE for 12 weeks was able to lower the increased bone turnover, which was reflected by the significant decreases in CTX and P1NP (Figure 1 and Figure 2).

An analysis of 3D bone microarchitecture by micro-CT showed that the alveolar bone in RDE- or E2-treated rats had less bone loss than the OVX group, which was reflected by the significant change in BV/TV, Tb.Th, Tb.N, Tb.Sp and SMI. E2 had a more potent anti-osteopenic effect on alveolar bone than RDE (Table 2). The histological observation of alveolar bone (Figure 4) confirmed the results of micro-CT assessment (Figure 3).

The results from the assays of BMD, micro-CT, bone turnover biomarkers and histological observation showed that RDE had a substantial anti-osteopenic effect on alveolar bone.

To explore the mechanism of the anti-osteopenic effect of RDE on alveolar bone, we screened the differential expression of genes by microarray and found key pathways with IPA. In our study, we succeeded in identifying 383 genes, which were differentially expressed in the alveolar bone of the RDE group compared to the OVX group. Table 3 shows the top five canonical pathways from the hundreds of canonical pathways (*p* < 0.05). Of the top five pathways, two pathways were related to bone metabolism. From Figure 5, we observed that the Wnt/β-catenin signaling pathway in osteoblasts and p38 MAPK signaling pathway in osteoclasts were downregulated after the RDE treatment.

The canonical Wnt/β-catenin signaling pathway is involved in osteoblast proliferation and bone matrix formation, stabilization and mineralization [27,28] and is initiated by the binding of Wnt ligands to the complex compromised of frizzled homolog proteins (FZDs) and low-density lipoprotein receptor-related proteins 5/6 (LRP5/6) [29]. The resultant signals prevent β-catenin phosphorylation by a multiprotein complex composed of adenomatous polyposis coli (APC), glycogen synthase kinase 3β (GSK-3β), casein kinase 1 (CK-1) and axins, causing its proteasomal degradation. The β-catenin associates with T-cell factor (TCF)/lymphocyte enhancer transcription factors (LEF) to activate target genes [30]. The secreted frizzled-related proteins (sFRP1 to sFRP5) are a family of soluble proteins that are structurally related to FZDs. Because of their homology with the Wnt-binding domain on the FZDs, SFRPs were immediately characterized as antagonists that bind to Wnt proteins to prevent signal activation [31].

In the canonical Wnt/β-catenin signaling pathway, the TCF/LEF family transcription factors (e.g., TCF3) played a pivotal role in promoting bone-specific gene expression, such as Runt-related gene 2 (RUNX2) [32]. RUNX2 is a multifunctional transcription factor that controls skeletal development by regulating the differentiation of osteoblasts and the expression of many extracellular matrix protein genes [33]. During osteoblast differentiation, RUNX2 upregulates the expression of bone matrix protein genes, including SPP1, also known as OPN [34]. SPP1 is an early marker of periodontal tissue regeneration, which is temporally and spatially associated with intensive cell proliferation and migration in the osteogenic cell population [35]. An *in vitro* study showed that diosgenin could induce RUNX2-regulated osteopontin protein expression in osteoblasts at low concentrations, and again, this decreased at high concentrations [36].

We found that gene expressions of *Wnt7a*, *Fzd2*, *Tcf3* and *Spp1* in the alveolar bone were downregulated, and *Frzb*, *Sfrp2* and *Sfrp4* were upregulated after treatment with RDE (Figure 6). This regulation of gene expression led to an inhibitory effect on the Wnt/β-catenin signaling, which plays a pivotal role in osteoblastogenesis. Our qRT-PCR analyses of *Wnt7a*, *Frzb*, *Fzd2*, *Tcf3*, *Spp1*, *Sfrp2* and *Sfrp4* expressions (Figure 6a–g) and western blotting analysis of Tcf-3 (Figure 7) convinced us that RDE could decrease the high bone formation in the alveolar bone of OVX rats, which results from ovariectomy by attenuating the canonical Wnt/β-catenin signaling, and that the results of the microarray and IPA were credible.

MAPK family members are classified into three groups: the ERK, JNK and p38 MAPK groups. Phosphorylation of p38 MAPK (p38α coded by *Mapk14*, p38β coded by *Mapk11* and p38γ coded by *Mapk12*) by MAPK kinase (MKK) 3/6 results in p38 MAPK activation. Activated p38 MAPK then phosphorylates transcription factor ATF2, which, in turn, induces target gene transcription [37,38]. p38 MAPK is primarily activated within cells involved in the inflammatory process, which, in turn, induces the synthesis of key inflammatory mediators, such as tumor necrosis factor α (TNF-α), interleukin (IL)-1, IL-6, IL-8 and cyclooxygenase-2, either via direct gene transcription activation or messenger RNA stabilization. In the context of bone, p38 MAPK occupies a central role in the regulation of IL-1 and TNF-α-signaling networks and NF-κB ligand (RANKL)-induced osteoclastic bone resorption [39]. Several studies showed that p38 MAPK signaling is involved in osteoclast differentiation, which is responsible for periapical alveolar bone resorption, and that the p38 MAPK inhibitor could inhibit active alveolar bone loss in a rat periodontitis model [40,41].

We found that the expressions of *Il1rn* (upregulated) and *Mapk14* (downregulated) in the alveolar bone were regulated after treatment with RDE (Figure 5). Our qRT-PCR analyses of *Il1rn* and *Mapk14* expressions (Figure 6i,j) and western blotting analysis of p38α (Figure 7) convinced us that RDE could decrease high bone resorption in the alveolar bone of OVX rats, which results from ovariectomy by attenuating p38 MAPK signaling, and that the results of the microarray and IPA were credible.

After the treatment of RDE, the expressions of *Wnt7a*, *Fzd2*, *Tcf3*, *Spp1* and *Mapk14* in the alveolar bone were down regulated, while the expressions of *Frzb*, *Sfrp2*, *Sfrp4* and *Il1rn* were up regulated. On the one hand, the regulation of *Wnt7a*, *Frzb*, *Fzd2*, *Tcf3*, *Spp1*, *Sfrp2* and *Sfrp4* could inhibit osteoblastogenesis via attenuating Wnt/β-catenin signaling. On the other hand, the regulation of *Il1rn* and *Mapk14* could inhibit osteoclastogenesis via reducing p38 MAPK signaling. Our qRT-PCR or western blotting analyses of these gene or protein expressions convinced us that RDE could decrease the excessively high bone formation and the bone resorption synchronously in the OVX rat alveolar bone to a certain extent. This inhibitory effect of RDE was associated with the Wnt/β-catenin signaling and p38 MAPK signaling. Furthermore, because rats in the RDE group had higher bone mass than those in the OVX group, we inferred that RDE had a more potent inhibitory effect on bone resorption than bone formation.

In this research, we only explored the anti-osteopenic effect of RDE in an animal model. A line of studies had to be performed before RDE became a promising alternative preventive and therapeutic agent for relieving alveolar bone loss of postmenopausal women.

## 5. Conclusions

RDE may inhibit ovariectomy-induced alveolar bone loss in rats. The mechanism for the anti-osteopenic effect of RDE may lie in the simultaneous inhibition of both bone formation and bone resorption that occurs following regulation of Wnt/β-catenin signaling and p38 MAPK signaling. Our study provides evidence that the aqueous extract of *Rhizoma Dioscoreae* may have potential use as an oral drug for treating alveolar bone loss in postmenopausal females.

## Supplementary Files

Supplementary File 1
